# Editorial: Integrating Healthcare Worker Wellbeing and Clinical Practice: Sisyphean Task or Reconcilable Duty?

**DOI:** 10.3389/fpsyg.2022.863896

**Published:** 2022-03-22

**Authors:** Anthony Montgomery, Renato Pisanti, Efharis Panagopoulou

**Affiliations:** ^1^University of Macedonia, Thessaloniki, Greece; ^2^University Niccolò Cusano, Rome, Italy; ^3^Aristotle University of Thessaloniki, Thessaloniki, Greece

**Keywords:** burnout, healthcare worker wellbeing, patient safety, quality of care, patient experience

Health care reforms in the recent decades have created a state of never-ending change that is stressful for health care workers (OECD., 2019). Health professionals are particularly affected by economic constraints in healthcare systems that challenge their ability to provide high-quality care according to their professional standards (Edwards and Burnard, 2003; O'Connor et al., 2020).

It is widely accepted that our healthcare employees are under increasing levels of stress, while the demand to provide safer and efficient care is growing in tandem (Montgomery et al., 2020). We run the risk of asking our healthcare workers to do more with less.

The aforementioned is exacerbated by solutions to the problems of healthcare worker wellbeing that valorise individual over organizational and system level approaches (Montgomery et al., 2019). Not surprisingly, such continuous depredation of our health professionals will ultimately be visited upon patients (Teoh and Hassard, 2020). There is a growing call for the meaningful engagement of patients and the public in healthcare delivery and design (Boger et al., 2015).

This Research Topic explores the antecedents of well being among health care workers: five articles specifically address various facets of this ubiquitous phenomenon and are the focus of our analysis. Three articles focus on individual protective factors, such as psychological flexibility and specific character strengths (Holmberg et al.; Huber et al.; Kachel et al.); and two articles focus on the role of organizational positive dimensions such as perceived organizational support and organizational cultures (Ramos et al.; Sarwar et al.). The articles are of interest to us because they highlight how improving healthcare worker wellbeing needs to be rooted in both individual and organizational solutions. This is important because creating healthy work environments in hospitals and allied healthcare organizations involves us moving the focus away from a physician-centric approach toward one that asks what can be done for the modern healthcare setting to enhance the ability of all workers in healthcare to thrive (Maeda and Socha-Dietrich, 2021).

In the first article on psychological flexibility as an individual protective factor, among intensive care medical staff, Holmberg et al. show the indirect effect of psychological flexibility on the relationship between distress and work engagement. Furthermore, in a longitudinal subsample, authors reported a significant association between an increase in psychological flexibility and increase in work engagement. Therefore, psychological flexibility can be considered an addressable and meaningful target for interventions aimed at improving work engagement (Holmberg et al.; page 8).

Two studies analyzed (Huber et al.; Kachel et al.) the impact of character strengths on well being among Austrian health care professionals. Kachel et al. investigated whether there are specific character strengths among medical professionals that are particularly relevant to work-related well being (thriving, work engagement, and burnout dimensions). Results from quantitative and qualitative data suggested that beneficial character strengths for work-related well being may be driven by different work experiences, professional understandings, generational beliefs, or social expectations.  Huber et al. carried out a longitudinal study among medical students to examine potential protective effects of the applicability of signature character strengths over time. They found significant positive effects of well being dimensions (thriving and personal well being) on signature strengths' applicability at later time points. Moreover, authors found that work engagement showed a significant mediator role between the applicability of signature character strengths and well being dimensions (thriving, subjective well being and psychological well being). In both articles, authors described interesting practical implications. Tailored interventions could focus on the ability to cultivate a sense of meaning in work, or implement mindfulness-based strengths practice into medical curricula and training of physicians.

Regarding organizational level, Ramos et al. addressed the key organizational culture factors in order to project and to implement interventions to reduce burnout and occupational stress in an inpatient clinical department at a major European Hospital. Authors described an intervention conducted under the lens of the Action Research (AR) methodology. The specific AR model for the health context was developed by the researchers of the European Project ORCAB—*Improving quality and safety in the hospital: the link between organizational culture, burnout and quality of care—*and it included five cyclical stages: (1) problem identification, (2) action planning, (3) implementation, (4) evaluation, and (5) reflection. Furthermore, the authors highlighted the main obstacles dealt with the research team. Although, the health care workers proposed to develop interventions focussed on the emotional labor, the hospital' hierarchy blocked these possibilities.

Sarwar et al. argued that understanding the reasons why nurses engage in service sabotage behaviors—where personnel deliberately trouble the rightful interests of a customer/patient—may provide useful insights. Authors applied the conservation of resources (COR) theory and equity theory, hypothesizing: (a) workplace ostracism (WPO) (i.e. employee's perceptions of being ostracized) is positively associated with customer service sabotage (CSS); (b) psychological stress mediates the positive relationship between WPO and CSS; (c) perceived organizational support (POS) moderates the indirect association between WPO and CSS through psychological stress such that the association will become weaker in the presence of high POS and vice-versa. Confirmatory factor analysis supported the mediator and moderator effects hypothesized by the authors.

Together, the articles in this Research Topic contribute to our knowledge about healthcare worker well being offering new insights about the interplay among individual, group and organizational level of analysis. Collectively, they recommend the development of an organizational culture that encourages healthcare professionals to keep a balance between taking care of others and taking care of themselves, and their personal well being. Moreover interventions, designing work practices that increase the opportunities of social connectivity and promoting individual skills of coping, should be promoted and implemented with the involvement of hospital management and of the leaders of each teamwork.

## Author Contributions

All authors listed have made a substantial, direct, and intellectual contribution to the work and approved it for publication.

## Conflict of Interest

The authors declare that the research was conducted in the absence of any commercial or financial relationships that could be construed as a potential conflict of interest.

## Publisher's Note

All claims expressed in this article are solely those of the authors and do not necessarily represent those of their affiliated organizations, or those of the publisher, the editors and the reviewers. Any product that may be evaluated in this article, or claim that may be made by its manufacturer, is not guaranteed or endorsed by the publisher.
